# Biomolecular Mechanisms of Cardiorenal Protection with Sodium-Glucose Co-Transporter 2 Inhibitors

**DOI:** 10.3390/biom12101349

**Published:** 2022-09-22

**Authors:** Francesca Romana Prandi, Lucy Barone, Dalgisio Lecis, Martina Belli, Domenico Sergi, Marialucia Milite, Stamatios Lerakis, Francesco Romeo, Francesco Barillà

**Affiliations:** 1 Division of Cardiology, Department of Systems Medicine, Tor Vergata University, 00133 Rome, Italy; 2 Department of Cardiology, Mount Sinai Hospital, Icahn School of Medicine at Mount Sinai, New York, NY 10029, USA; 3 Cardiovascular Imaging Unit, San Raffaele Scientific Institute, 20132 Milan, Italy; 4 Faculty of Medicine, Unicamillus-Saint Camillus International University of Health and Medical Sciences, 00131 Rome, Italy

**Keywords:** SGLT2 inhibitors, diabetes mellitus, cardiorenal protection, biomarkers

## Abstract

Diabetes mellitus (DM) is a metabolic disorder characterized by chronic hyperglycemia and associated with an increased risk of morbidity and mortality, primarily from cardiovascular and renal diseases. Sodium-glucose cotransporter 2 inhibitors (SGLT2-Is) are novel drugs for the treatment of type 2 DM and heart failure (HF). SGLT2-Is mediate protective effects on both the renal and cardiovascular systems. This review addresses the current knowledge on the biomolecular mechanisms of the cardiorenal protective effects of SGLT2-Is, which appear to act mainly through non-glucose-mediated pathways. Cardiorenal protection mechanisms lead to reduced chronic renal disease progression and improved myocardial and coronary endothelial function. Concomitantly, it is possible to observe reflected changes in biomarkers linked with diabetic kidney disease and HF.

## 1. Introduction

Diabetes mellitus (DM) is a glucose metabolism disorder characterized by chronic hyperglycemia, resulting from a deficit in insulin production and/or action. DM represents one of the most common health problems worldwide; it affects 537 million people, with 10.5% prevalence worldwide in 2021 [1], and it is associated with an increased risk of morbidity and mortality. Approximately 6.7 million adults are estimated to have died globally from diabetes or from its complications in 2021 (12.2% of all deaths) [2].

Type 1 diabetes mellitus (T1DM) accounts for approximately 5–10% of all patients with DM, and it results from pancreatic beta cell dysfunction with reduced insulin secretion, while type 2 DM (T2DM) is related to insulin resistance and accounts for 90–95% of all diabetic patients [3].

Hyperglycemic damage on vascular endothelial cells, leading to endothelial dysfunction, represents the main initiating factor in the pathogenesis of diabetic micro- and macrovascular complications, in both T1DM and T2DM patients. In addition, T2DM also causes insulin resistance (IR), which is another determinant for endothelial dysfunction [4]. IR is usually defined as decreased sensitivity to insulin and it plays an important pathophysiological role in the development of prediabetes and T2DM [5]. The onset of insulin resistance typically begins years before diabetes and even before prediabetes [6]. Skeletal muscle tissue insulin resistance can be considered the primary defect, present decades before impaired β-cell function [7]. Furthermore, in a study by Cerasi et al., it was observed that glucose-induced insulin release by pancreatic β-cells was decreased in diabetic and prediabetic subjects [8]. Combining the continued increase in IR with a decrease in β-cell function, blood glucose levels eventually become unregulated, and prediabetes then develops into overt diabetes.

Diabetic macro- and microvascular complications lead to cardiovascular and renal disease, which are the most common causes of death in diabetic patients. T2DM is a major cause of chronic kidney and cardiovascular diseases [9]. In clinical trials for patients with T2DM, the prevalence of heart failure (HF) ranged from ~10 to 30% [10,11,12], while, in trials of patients with chronic HF, the prevalence of T2DM was nearly 30% regardless of etiology HF [13,14,15]. In T2DM patients, 10% of deaths are due to the onset and progression of renal failure [16], and diabetic nephropathy accounts for 26.9% of the overall chronic kidney disease (CKD) hospitalizations [17].

The improved mortality outcomes achieved with some classes of hypoglycemic agents are largely independent of their glycemic effects [18].

Sodium-glucose co-transporter 2 (SGLT2) is a family of glucose transporter proteins localized in the proximal tubule of the nephron, responsible for the majority of filtered glucose and sodium reabsorption, and SGLT2 inhibitors (SGLT2-Is) are novel drugs for the treatment of T2DM and heart failure [19,20].

SGLT2-Is reduce the renal threshold for glucose excretion, resulting in an increase in urinary glucose excretion and a decrease in blood glucose levels. This reduces glucotoxicity and improves whole-body β-cell function and insulin sensitivity [21,22].

The precursor of SGLT-Is, the phlorizin, was isolated from the root of the apple tree in 1835, and its glycosuric properties were identified a century later [23,24].

In the last decade, several SGLT2-Is have been developed as derivatives of this drug, including dapagliflozin, canagliflozin, empagliflozin, ertugliflozin, ipragliflozin, luseogliflozin, and tofogliflozin. In addition to their structural differences, these agents have different half-lives and selectivity for SGLT2 co-transporters. The United States Food and Drug Administration and the European Union currently have approved only the first four SGLT2-Is [25]. In contrast, ipragliflozin, tofogliflozin, and luseogliflozin have been authorized in Japan [26,27].

The protective effect of SGLT2-Is on the reduction of acute and chronic cardiovascular (CV) events is well known [28]. Therefore, early clinical trials of SGLT2-Is focused on improving glucose plasma levels and other diabetes-related effects. However, the CV benefits observed in several studies generated considerable interest in their therapeutic benefits beyond glycemic control, and a number of cardiovascular outcome studies have been conducted over the past six years. Unexpectedly, these drugs have shown a reduction in the risk of cardiovascular disease in general, with particularly marked effects on CV death and hospitalization for HF [29,30].

A number of large clinical trials have been conducted to evaluate the safety and efficacy of SGLT2-Is in patients with diabetes and established vascular disease, multiple cardiovascular risk factors, or renal failure and in patients with established HF and reduced ejection fraction, with and without T2DM [31]. The most important results of the EMPA-REG OUTCOME (Empagliflozin Cardiovascular Outcomes Event Trial in Type 2 Diabetes Mellitus Patients—Removing Excess Glucose) trial showed that T2DM patients who were at high risk for CV disease had early reductions in major CV and renal sequelae [32]. This included a significant reduction in CV death and hospitalization for HF in patients treated with empagliflozin. Subsequent large trials with other SGLT2-Is, such as canagliflozin (CANVAS (Canagliflozin Cardiovascular Assessment Study) [33] and CREDENCE (Canagliflozin and Renal Outcomes in Type 2 Diabetes and Nephropathy) trials [34]) and dapagliflozin (DECLARE-TIMI 58 (Dapagliflozin and Cardiovascular Outcomes in Type 2 Diabetes) trial [35]), confirmed these observations in a broader population of primary and secondary prevention patients [36,37]. O’Hara et al., in a recent review, showed that the administration of dapaglifozin in non-diabetic patients may prevent the onset of overt diabetes mellitus [38]. This observation came from the results of a pre-specified pooled analysis of the DAPA-CKD [39] and DAPA-HF [30] trials and raises the possibility of an expanded target patient population.

In addition, SGLT2-Is showed a lower risk of CKD, even in non-diabetic patients [29]. Research to date to identify the mechanisms of action of SGLT2-Is has yielded impressive results. The lack of consistent glucose dependence of cardiorenal protection, with no significant difference between subjects with and without T2DM and across levels of glucose control in T2DM patients, suggested a predominant role of non-glucose-mediated pathways [29,30].

In the last decade, recent observations have revealed a surprisingly wide variety of beneficial effects.

In the kidney, SGLT2-Is act by restoring sodium/fluid homeostasis; they have pleiotropic effects on calcium/phosphate homeostasis, magnesium levels, glomerular tubular feedback, and energy metabolism. In the heart, they modulate the cardiac sodium-hydrogen exchanger. Other effects include systemic metabolic and hemodynamic adjustments, the attenuation of mitochondrial dysfunction, oxidative stress, and inflammation, and the stimulation of autophagy [40].

**Figure 1:**
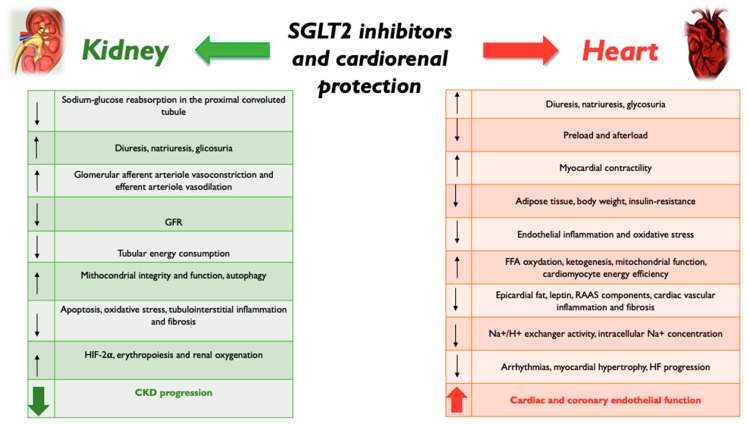
SGLT2-Is cardiorenal protection mechanisms.

**Figure 2:**
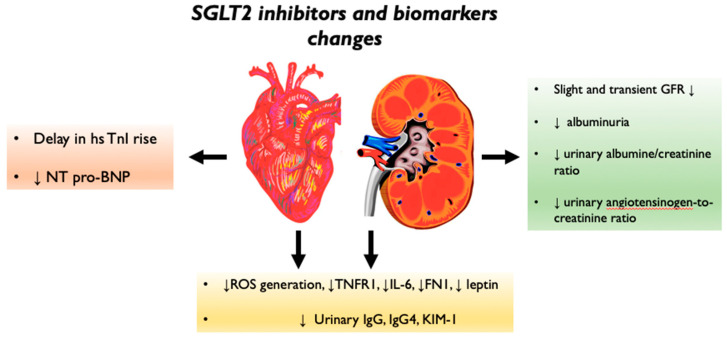
SGLT2 inhibitor and biomarker changes.

In this review, the authors summarize the current state of knowledge of the biomolecular mechanisms of SGLT2 inhibition in cardiorenal protection (Figure 1) and the changes in biomarkers linked with diabetic kidney disease (DKD) and HF observed with the use of SGLT2-Is (Figure 2).

## 2. SGLT2 Inhibition and Kidney Protection

In healthy adult men, maximal glucose tubular transport (TmG) is approximately 375 mg/min, which correlates with 300 mg of glucose per day [41,42]. During normoglycemic and hypoglycemic conditions, filtered glucose is completely reabsorbed at the proximal convoluted tubules by means of SGLT2, as the glucose filtration rate is lower than TmG. Glycosuria appears when glycemia exceeds the threshold level of 180 mg/d. Underlying this is the action of SGLT2, located below Bowman’s capsule in the S1 segment of the proximal tubule, and sodium-glucose co-transporter 1 (SGLT1), located in the S2–S3 segment below [43]. Around 80–90% of filtered glucose is reabsorbed by SGLT2, and the remainder by SGLT1. SGLT2 has high transport capacity, but lower affinity for glucose, unlike SGLT1. Patients with T2DM present higher expression of SGLT2s in the proximal tubule compared to healthy individuals [44].

During hyperglycemia, as glucose and sodium reabsorption are coupled, increased glucose reabsorption leads to increased sodium reabsorption, which is responsible for hypertension in diabetic subjects. The increased extracellular fluid volume resulting from the hyperabsorption of sodium also causes an atrial natriuretic peptide rise and vasodilatation of the glomerular afferent arteriole. In addition, a low sodium concentration detected at the macula densa activates the renin–angiotensin–aldosterone system (RAAS), with increased production of angiotensin II (ATII) and vasoconstriction of the efferent arteriole [45]. Vasodilation of the afferent arteriole by nitric oxide, adenosine, and prostanoids and vasoconstriction of the efferent arteriole by ATII result in increased endoglomerular filtration pressure with glomerular hyperfiltration, which damages the mesangial structure and causes inflammation, fibrosis, renal damage, and albuminuria [46]. SGLT2 inhibition leads to an increased concentration of sodium chloride in the tubular fluid, which may trigger a cascade that reduces the GFR by afferent glomerular arteriole constriction. SGLT2-I-mediated diuresis and natriuresis also reduce the concentration of circulating natriuretic peptides, which contributes to vasoconstriction of the efferent arteriole [45]. Moreover, the macula densa sensing of an increased sodium concentration activates the tubuloglomerular feedback, which causes vasoconstriction of the afferent arteriole and suppresses renin production by juxtaglomerular cells, reducing RAAS system activation and enhancing efferent glomerular arteriole vasodilation, which further reduces GFR and glomerular hyperfiltration. SGLT2-Is reduce active tubular transport work and, thereby, reduce the energy demand and oxygen consumption in the kidney [47], showing that it is precisely reduced cortical oxygenation that predicts the progressive decline in renal function. Thus, by reducing glomerular hyperfiltration and hyperglycemia, SGLT2 inhibition also decreases albuminuria, tubular growth, and tubulo-interstitial inflammation. SGLT2-I cardiovascular outcome trials in diabetes mellitus patients demonstrated that empagliflozin, canagliflozin, and dapagliflozin reduce hyperfiltration at the onset of therapy and slow the decline in estimated glomerular filtration rate (eGFR) in the long-term. This might be due to an improvement in mitochondria integrity and function, tubular energy, and autophagy. Darshi M et al. showed that diabetes increases the urinary ratio of lactate to pyruvate, thus inducing an increase in glycolysis at the expense of mitochondrial oxidation [48], and they demonstrated that this dangerous process was reversed by SGLT2-I administration. In addition, dapagliflozin treatment improves mitochondrial function in diabetics, increasing urinary metabolites linked to mitochondrial metabolism compared with a placebo, suggesting that SGLT2 inhibition may improve mitochondrial function in diabetes [49]. Lee et al. showed that empagliflozin treatment improved mitochondrial fragmentation and enhanced renal proximal tubule cell autophagic activity under hyperglycemia by involving the AMP-activated protein kinase (AMPK) and the mammalian target of rapamycin (mTOR) signaling pathways, resulting in reduced apoptosis and tubulo-interstitial fibrosis [50], so empaglifozin improved tubular mitochondrial dynamics and increased autophagic activity. Autophagy is a mechanism that allows healthy renal tubules and podocytes to maintain their structural and functional integrity by counteracting oxidative stress. In the diabetic kidney, excessive oxidative stress is not adequately limited [51] as the autophagy process is impaired, resulting in the dysfunction and death of podocytes and tubular cells [52]. The last mechanism responsible for the nephroprotective effect of gliflozins is the stimulation of erythrocytosis by acting on hypoxia-inducible factors (HIFs; specifically, HIF-1α and HIF-2α). HIF-2α is the isoform that is responsible for erythropoietin synthesis [53]. This action is related to the expression of HIF2α in specialized peritubular interstitial cells in the kidney, although HIF-2α also mediates erythropoietin synthesis in the liver [54,55]. In contrast, although HIF-1α can influence erythropoiesis if HIF-2α signaling is impaired, the diabetic kidney is dominated by oxidative stress and hypoxia, with activation of HIF-1α and inhibition of HIF2α. SGLT2-Is reduce renal hypoxia through activation of HIF-2α, resulting in increased erythropoiesis (Figure 1).

Dapaglifozin also exerts nephroprotection by an epigenetic mechanism, preserving the renal vasodilating capacity through a reduction in miRNA-27b expression [56].

## 3. SGLT2 Inhibition and Heart Protection

While the nephroprotective action has solid evidence, the mechanisms responsible for SGLT2-I-related cardioprotection are still not well understood. Today, gliflozins represent one of the “four pillars” of therapy for reduced ejection fraction heart failure (HFrEF), to be introduced immediately in combination with ARNI (or ACE inhibitors/sartan), beta-blockers, and mineralocorticoid antagonists in recommendation class IA. The first published study, DAPA-HF [57], evaluated the efficacy of dapaglifozin vs. placebo on a primary endpoint of worsening HF, i.e., hospitalization, and cardiovascular death in 4744 patients with HFrEF, regardless of the presence of diabetes. The primary endpoint was reduced in 26% of patients. Similar results were recently obtained with empaglifozin in the Emperor Reduced study [58], which demonstrated a superior reduction in adverse events of empaglifozin to placebo. Among the cardioprotective effects are certainly the vascular and hemodynamic ones. The glycosuric action is associated with a diuretic action [59,60], resulting in increased reabsorption of interstitial edema and a reduction in preload and afterload with positive hemodynamic effects. According to the Frank–Starling law, the cardiomyocyte contractility is directly related to cell distension and preload, but overdistension impairs contractility and leads to a reduction in cardiac output. Therefore, natriuresis and diuresis associated with SGLT2 pathway inhibition may improve cardiac contractility by reducing the plasma volume, preload, and myocardial overdistension. The reduction in blood pressure and afterload induced by gliflozins represent another important mechanism that improves cardiac function [61]. Osmotic diuresis is responsible for the reduction in blood pressure values; in particular, treatment with SGLT2-Is is associated with a significant decrease in blood pressure—an average reduction of approximately 3–4.5 mmHg for systolic blood pressure and 1–2 mmHg for diastolic blood pressure compared to baseline levels [62]. The results of four placebo-controlled studies showed that canagliflozin causes a significant reduction in systolic blood pressure compared to placebo, with a dose-dependent effect (−4.00 mmHg at the 100 mg dosage and −5.0 mmHg with 300 mg) [63]. The same analysis showed that 67.1% and 69.9%, respectively, of patients treated with canagliflozin 100 and 300 mg reached the target value of systolic blood pressure <140 mmHg (32.2% and 39.8%, respectively, for the target value <130 mmHg). Canaglifozin has been shown to return the urine volume to pre-treatment levels after 3 months of treatment, although the blood pressure reduction persists [64], suggesting that, besides diuresis, also other mechanisms contribute to the reduction in arterial pressure, such as nephron remodeling, endothelial function improvement, arterial stiffness reduction, and loss of body weight related to caloric loss through glycosuria [45]. The glycosuria-related reduction in body weight and adipose tissue also leads to a reduction in insulin resistance [65]. In addition, there is a change in adipose tissue expression of adipokines and leptin with a reduction in inflammation and oxidative stress at the endothelial level [66]. Inhibiting proinflammatory–oxidative pathways, SGLT2-Is improve coronary endothelial function and enhance flow-mediated vasodilatation.

In patients with HF there is a decreased ATP availability with a shift from the mitochondrial oxidation of glucose to glycolysis. SGLT2 inhibition, on the other hand, increases free fatty acid oxidation, stimulating ketogenesis and shifting substrate use towards fat; this improves mitochondrial function, cardiomyocyte energy efficiency, and ventricular contractile performance [67,68]. In detail, SGLT2-Is are associated with increased ketogenesis as a consequence of the reduced renal glucose reabsorption [61]. Ketone bodies are produced in the liver and are utilized as energy-efficient substrates in extrahepatic tissue, including the heart and kidneys. β-Hydroxybutyrate (β-HB) is the predominant ketone body utilized in the heart; it is converted to acetoacetate via β-hydroxybutyrate dehydrogenase 1 and activated by the succinyl-CoA:3-oxoacid CoA transferase to form acetoacetyl CoA. Acetoacetyl-CoA undergoes a thiolysis process to form acetyl-CoA and then enters the tricarboxylic acid cycle to produce adenosine triphosphate (ATP) for cardiac contraction [69]. SGLT2-Is can raise the level of β-HB to around 0.6 mmol/L in patients with diabetes and to around 0.3 mmol/L in those without diabetes [67]. β-HB has been shown to have several hemodynamic effects, including a decrease in peripheral vascular resistance and filling pressures, a mild increase in heart rate, and an increased cardiac output; in addition, β-HB may have anti-inflammatory effects, through NLRP3 inflammasome inhibition and inflammatory cytokine production reduction, and may also act as an epigenetic modifier [70]. Empaglifozin ameliorated adverse cardiac remodeling and HF in a non-diabetic porcine model, by switching myocardial fuel utilization toward ketone bodies [71]. SGLT2-Is also reduce epicardial adipose tissue, which could result in the reduction of leptin and RAAS components, which are involved in cardiac vascular inflammation and fibrosis [72]. Empaglifozin, canaglifozin, and dapaglifozin exert their cardioprotective action also by inhibiting the cardiac Na^+^/hydrogen exchanger, leading to lower cytosolic Na^+^ and Ca^2+^ concentrations and increased mithocondrial Ca^++^ concentrations [73]. Increased Na^+^/hydrogen exchanger activity and cardiomyocyte intracellular Na^+^ concentrations have been associated with an increased risk of arrhythmias [74], myocardial hypertrophy, and HF aggravation (Figure 1) [75]. Dapaglifozin also has anti-inflammatory action through the inactivation of ROS and intracellular NLRP3 inflammasome activation [76].

The efficacy of these drugs in the improvement of cardiac function lies also in their epigenetic effects [4].

Solini et al. suggested novel mechanisms of cardioprotection associated with dapaglifozin treatment. This drug upregulates miRNA-30e-5p, which inhibits myocardiocyte autophagy and heart failure onset and reduces miRNA-199a-3p expression, which causes a reduction in cardiac PPARδ levels, thus restoring mitochondrial fatty acid oxidation and leading to an improvement in cardiac function in patients with HF [56].

## 4. Changes in Biomarkers Linked with Diabetic Kidney Disease and Heart Failure with SGLT2-Is

Several biomarkers linked to DKD and HF have been investigated in recent years in humans and animal models after the initiation of therapy with gliflozins (Figure 2).

DKD is a challenging complication in patients affected by type 1 or type 2 diabetes mellitus. To predict the onset of the feared end-stage renal disease in patients with DKD, several studies have shown that the most reliable prognostic biomarker is the current and past GFR trajectory [77,78]. Another important biomarker in DKD is albuminuria, which predicts the disease progression. However, the specificity and sensitivity of albuminuria to reveal ESRD and a decline in eGFR are very low [79]. Referring to SGLT2 inhibitors, it has been observed that after the initiation of therapy in humans, there is a transient and slight decrease in eGFR associated with a reduction in albuminuria by 30–50% [80]. The explanation of this phenomena seems to be due to the activation of the tubuloglomerular feedback, which induces a decrease in the intraglomerular pressure [81]. One of the most common biomarkers of renal function is serum creatinine [82]. The CREDENCE trial demonstrated for the first time that SGLT2 inhibition slows the advancement of DKD. This trial was focused on kidney function in patients with T2D, eGFR of 30–90 mL/min/1.73 m^2^, and a urine albumin-to-creatinine ratio (UACR) of 300–500 mg/g who underwent canaglifozin therapy. In patients treated with canaglifozin, the primary composite outcome of ESRD, doubling of serum creatinine or death from renal or CV causes, was reduced by 30% [83]. The urine angiotensinogen-to-creatinine ratio, a marker of intrarenal RAAS, declines in patients treated with SGLT2-Is [84].

SGLT2 inhibitors have also been associated with a reverse ventricular remodeling in patients with HF. This effect is associated with the hemodynamic changes of reduced preload and afterload, which SGLT2 inhibitors induce after the start of therapy. Regarding cardiac biomarkers, it has been observed that medical therapy with canaglifozin in patients with HF delays the rise in high-sensitivity Troponin I, commonly associated with myocardial damage [85]. Several studies showed that the reversal of ventricular remodeling induced by SGLT2 inhibitors decreases the serum NT pro-BNP, well known as a biomarker of HF. These data are associated with the improvement of dyspnea in patients with HFrEF [85,86].

Diabetes is associated with a chronic, systemic, and low-grade inflammation state that plays an important role in the pathogenesis of DKD and HF [87]. SGLT2 inhibitors yield anti-inflammatory properties, which mediate their cardiovascular and kidney-protective effects. It has been shown by specific biomarkers that the SGLT2 inhibitors’ anti-inflammatory activity is associated with a reduction in oxidative stress and advanced glycolytic end products (AGEs). This activity has been reflected in the systemic reduction of pro-inflammatory cytokine/chemokines. Kaneto et al. showed that chronic hyperglycemia in patients with diabetes enhances the generation of ROS through the production of AGEs, the increase of NOX activity, and the mitochondrial ROS generation [88]. It is well known that cardiovascular disease and kidney disease are linked to increased oxidative stress. Indeed, dysfunctional cardiomyocytes are associated with excessive mitochondrial ROS production, leading to impaired ventricular contractility [89]. Pre-clinical studies on diabetic mice showed that SGLT2 inhibition decreases myocardial ROS production and cardiac fibrosis. Moreover, Li et al. demonstrated that gliflozins decrease ROS generation in endothelial cells from human coronary arteries [66]. The antioxidant effects of SGLT2 inhibitors have been related to the reduction of intracellular glucose in renal proximal tubule cells [90].

A small number of studies investigated the cytokine and chemokine responses to SGLT2 inhibitors. Canaglifozin, as shown by Heerspink et al., in patients with T2DM, reduces the blood levels of TNFR1, IL-6, and FN1. It is then possible to hypothesize that canaglifozin therapy leads to an improvement in fibrosis and inflammation [91].

SGLT2 inhibitors can exert their anti-inflammatory activity by reducing leptin secretion. Garvey et al. showed that canaglifozin, compared to glimepiride, reduced serum leptin by 25% while increasing by 17% the levels of adiponectin, which is an anti-inflammatory adipokine. A 22% reduction in median serum IL-6 level has been associated with canaglifozin therapy with no significant changes in HbA1c, lipid levels, or patient weight [92]. Dapaglifozin treatment has been associated with a reduction in the urinary levels of IgG, IgG4, and KIM-1, which are biomarkers of glomerular and tubular damage. Morevoer, dapaglifozin has been associated with a reduction in IL-6 [93].

It is possible to declare that all these studies suggest the existence of anti-inflammatory properties of SGLT2 inhibitors within the kidney and the heart, thus mediating, in patients affected by T2D, improvements in CV and kidney disease.

## 5. Conclusions

SGLT2-Is are novel drugs for the treatment of T2DM and HF that target the SGLT2 transporter protein in the kidney. Although SGLT2-Is reduce the renal threshold for glucose excretion, resulting in a decrease in blood glucose levels, the cardiorenal protective effects mediated by SGLT2-Is appear to be mainly related to non-glucose-mediated pathways.

In the kidneys, SGLT2-Is reduce glomerular hyperfiltration and active tubular transport work, improving mitochondrial dynamics and autophagic activity, and reducing apoptosis, oxidative stress, inflammation, and fibrosis. Through activation of HIF-2α, they also increase erythropoiesis and reduce renal hypoxia.

Among the cardioprotective effects, SGLT2-Is determine preload and afterload reduction, with improved hemodynamics; adipose tissue, body weight, and insulin resistance decrease, with reduced oxidative stress and endothelial inflammation; ketogenesis increase, with improved mitochondrial function and cardiomyocyte energy efficiency; and Na+/H+ exchanger activity inhibition, with reduced intracellular Na+ concentration and arrhythmias, myocardial hypertrophy, and HF progression incidence.

Cardiorenal protection mechanisms are reflected by changes in biomarkers, including albuminuria reduction, a delayed rise in high sensitivity Troponin I, and reduced NT pro-BNP and pro-inflammatory biomarkers.

## Data Availability

No new data were created or analyzed in this study. Data sharing is not applicable to this article.
